# Novel pH-sensitive nanoformulated docetaxel as a potential therapeutic strategy for the treatment of cholangiocarcinoma

**DOI:** 10.1186/s12951-015-0066-8

**Published:** 2015-02-27

**Authors:** Nan Du, Lin-Ping Song, Xiao-Song Li, Lei Wang, Ling Wan, Hong-Ying Ma, Hui Zhao

**Affiliations:** The Second Department of Oncology, The First Affiliated Hospital of the General Hospital of the PLA, Beijing, 100048 China; Department of Medical, The First Affiliated Hospital of the General Hospital of the PLA, No. 51 Fucheng Road, Haidian District, Beijing, 100048 China

**Keywords:** Cholangiocarcinoma, Polymeric micelles, Docetaxel, Apoptosis, Cancer chemotherapy

## Abstract

**Background:**

Cholangiocarcinoma (CC) is one of the fatal malignant neoplasms with poor prognosis. The traditional chemotherapy has been resistant to CC and does not improve the quality of life. The aim of the present study is to investigate the potential of chondroitin sulphate (CS)-histamine (HS) block copolymer micelles to improve the chemotherapeutic efficacy of docetaxel (DTX).

**Results:**

pH-responsive property of CS-HS micelles was utilized to achieve maximum therapeutic efficacy in CC. In the present study, docetaxel-loaded CS-HS micelles (CSH-DTX) controlled the release of drug in the basic pH while rapidly released its cargo in the tumor pH (pH 5 and 6.8) possibly due to the breakdown of polymeric micelles. A nanosize of <150 nm will allow its accumulation in the tumor interstitial spaces via EPR effect. CSH-DTX effectively killed the cancer kills in a time- and concentration-dependent manner and showed pronounced therapeutic action than that of free drug at all-time points. CSH-DTX resulted in higher apoptosis of cancer cells with ~30% and ~50 of cells in early apoptosis quadrant when treated with 100 and 1000 ng/ml of equivalent drug. The micellar formulations showed remarkable effect in controlling the tumor growth and reduced the overall tumor volume to 1/5^th^ to that of control and half to that of free drug treated group with no sign of drug-related adverse effects. Immunohistochemical analysis of tumor sections showed that fewer number of Ki-67 cells were present in CSH-DTX treated group comparing to that of free DTX treated group.

**Conclusion:**

Our data suggests that nanoformulation of DTX could potentially improve the chemotherapy treatment in cholangiocarcinoma as well as in other malignancies.

## Introduction

Cholangiocarcinoma (CC) is one of the fatal malignant neoplasms which arise from epithelium of biliary tract with high rate of mortality and morbidity [1,2]. The CC constitutes the 3% of gastrointestinal cancers with ~15% of overall hepatic cancers [3]. The incidence of CC among Western countries is 1–2 cases per 100000 persons however East Asia has higher incidence of CC with ~8 cases per 1000 individuals [4]. The CC has poor prognosis rate with 5-year survival rate of less than 10% and has a steady increase in the incidence rate. Approximately, 50% of CC cases are diagnosed at unresectable stage, as the symptoms are largely unknown at initial stages [5,6]. At present, surgical resection of CC tumor is the main treatment option for advanced stage tumor. However, surgical biliary bypass often causes serious postoperative complications and increases the morbidity rate. Additionally, palliative therapies such as endoscopic stent, radiation therapy, photodynamic therapy, and chemotherapy are employed to treat the CC [7]. Among all, chemotherapy is regarded as the adjuvant or main alternative treatment to CC, however traditional chemotherapy is reported to be resistant to CC and does not improve the quality of life [8,9]. Therefore, we need an effective therapeutic strategy that can overcome the limitation of conventional treatment modality and improve the chemotherapeutic effect in CC.

Nanotechnology-based drug delivery system has been reported to improve the pharmacological and anticancer property of chemotherapeutic drugs [10]. Specifically, fenestrated endothelium and heavy blood flow will allow the nanoparticles to be taken by the liver. This process can be accelerated by enhanced permeability and retention effect (EPR) that will allow the preferential accumulation or passive targeting of nanocarriers to the leaky vasculature of tumor tissues [11]. Importantly, the delivery carrier can be made responsive to the local microenvironment of tumor. The physiological pH of blood is ~7.2 while the pH of extracellular spaces around tumor is 6.8 and endolysosomes of cancer cells was very acidic (pH < 6) [12].

In this regard, block copolymer-based nanosized micelles have attracted significant attention as a promising delivery system towards cancer therapy [13]. Importantly, pH-responsive anticancer drug delivery has many benefits including high accumulation in tumor tissues, long blood circulation, limited release in physiological conditions, and utilizing EPR effect [14]. In the present study, we have conjugated chondroitin sulphate (CS) with histamine (HS) to form pH-responsive nanomicelles that can enhance the cancer cell killing effect. CS is a hydrophilic compound with excellent biocompatibility and biodegradability that made it an excellent choice for in vivo applications. CS is a vital structural component of cartilage and connective tissues. CS has been reported to target cancer cells by binding to the hyaluronic acid receptors expressed on the malignant cells and internalized actively. HS on the other hand was selected due to its imidazole ring characteristics [15]. The imidazole ring has a lone pair of electron on nitrogen that gives it amphoteric nature to protonate and deprotonate [16].

Docetaxel (DTX), is regarded as one of most effective chemotherapeutic agent for the cancer treatment. DTX is a typical microtubule inhibitor that binds with the microtubule assembly of cancer cells and prohibits its cell proliferation [17]. DTX is effective against wide range of cancers including ovarian, breast, head/neck, lung cancers, and liver cancers. Despite its promising clinical potential, severe side effects such as bone marrow suppression, hypersensitivity reactions, and peripheral neuropathy became a major obstacle. Additionally, poor water solubility and poor bioavailability limited its clinical application to a great extent [18].

Therefore, main aim of the present study was to load DTX in CS-HS-based nanomicelles and to utilize the pH-responsive property to achieve maximum therapeutic efficacy in Cholangiocarcinoma. The physicochemical characteristics of DTX-loaded CS-HS micelles (CSH-DTX) were studied in terms of size and release kinetics. In vitro cytotoxicity assay and apoptosis assay of free drug and CSH-DTX was studied in QBC939 adenocarcinoma cells. Antitumor efficacy of CSH-DTX was studied in xenograft nude mice and immunohistochemical studies were performed to evaluate its systemic performance.

## Results and discussion

**Figure 1:**
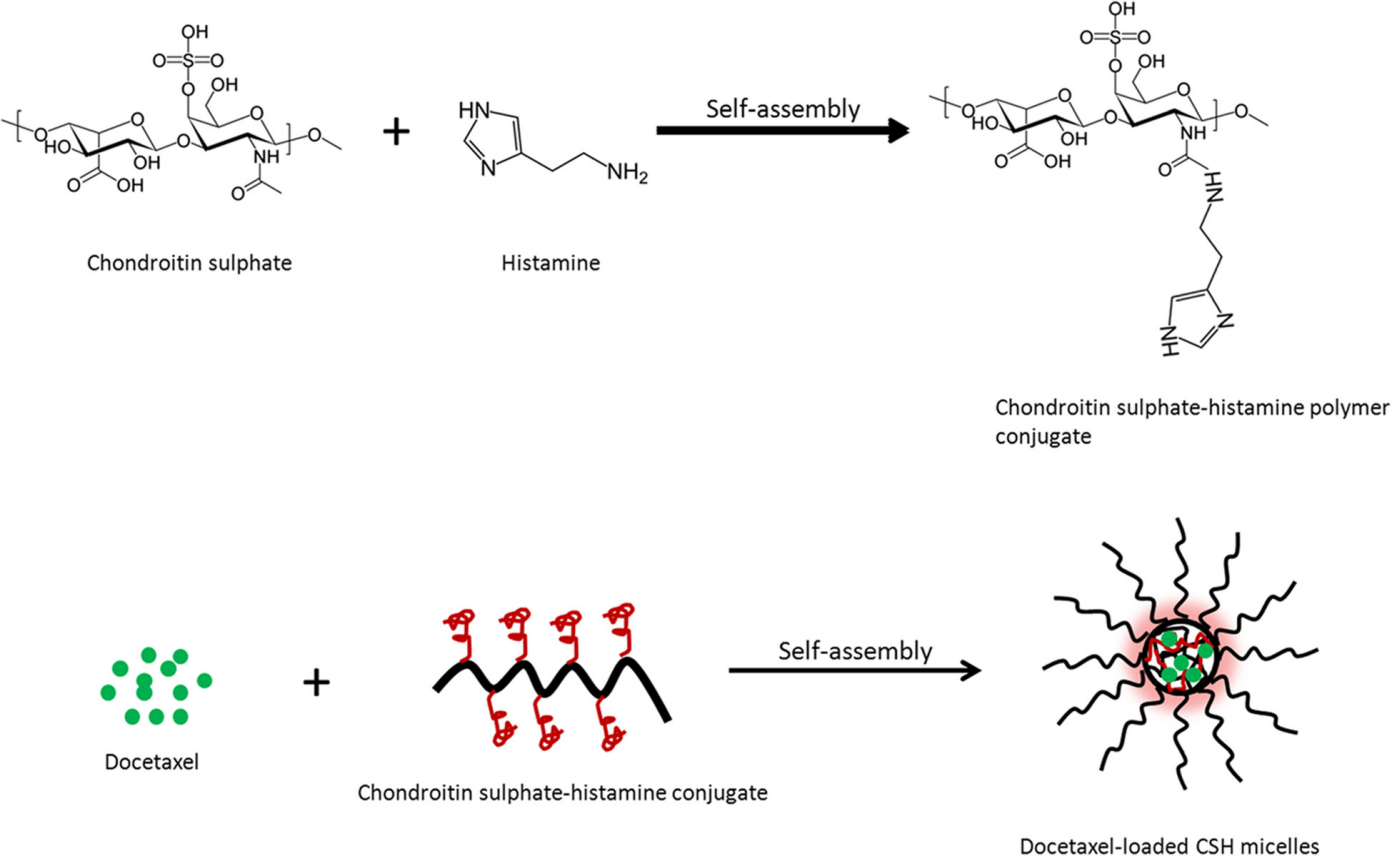
**Schematic representation of conjugation of chondroitin sulphate (CS)-histidine (HS) via chemical reactions.** Schematic illustration of self-assembly of docetaxel (DTX) and CS-HS conjugate into polymeric micelles.

Cholangiocarcinoma (CC) which arises from epithelium of bialy tract is one of the fatal malignant neoplasms with high rate of mortality and morbidity. At present, conventional chemotherapy is the main treatment option; however it does not improve the quality of patient life [2,4]. In this regard, nanotechnological solutions have been reported to improve the therapeutic performance of anticancer drugs. Importantly, a pH-responsive strategy would increase the accumulation in tumor tissues, extend blood circulation, and effectively improve the overall chemotherapeutic efficacy. In the present study therefore, we have conjugated chondroitin sulphate (CS) with histamine (HS) to form pH-responsive nanomicelles that can enhance the cancer cell killing effect. DTX, a typical microtubule inhibitor has been selected in this study as an anticancer drug to improve its therapeutic efficacy against CC [18]. Since the therapeutic application of DTX is hindered by its limited solubility and systemic toxicity, in the present study, DTX was loaded into CS-HS conjugate based polymeric micelles. DTX and CS-HS block copolymer when dissolved in water, hydrophobic and hydrophilic part self-assemble to form a drug loaded micelles (Figure 1). The so formed micelle (CSH-DTX) has numerous advantages including pH-sensitive drug via protonation of histidine residue, high loading efficiency, and potential clinical translational ability.

### Preparation and characterization of DTX-loaded micelles

**Figure 2:**
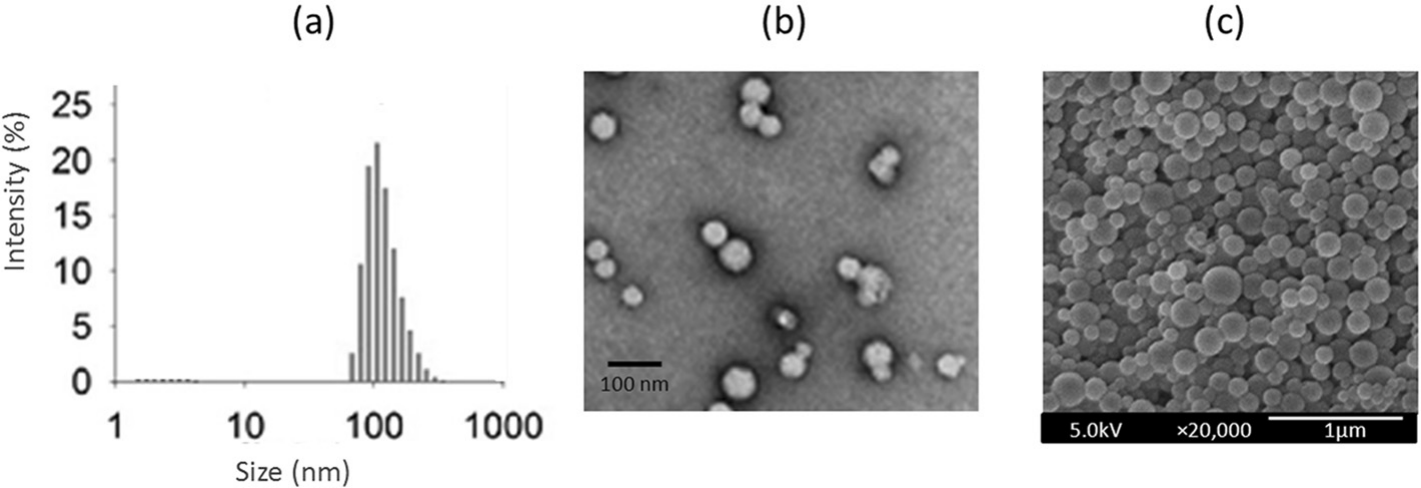
**(a) Typical size distribution analysis of CSH-DTX by dynamic light scattering technique (b) transmission electron microscope (TEM) imaging of CSH-DTX (c) scanning electron microscope (SEM) imaging of CSH-DTX.**

Physicochemical characterization of polymeric micelles was carried out in terms of particle size and polydispersity index. The particle size and PDI of CSH-DTX was measured by dynamic light scattering technique. The average size of CSH-DTX was observed to be around 110 nm with a fairly uniform dispersion of NP (PDI ~ 0.15) (Figure 2a). It has been previously reported that micelles size less than <200 nm could be preferentially accumulated in the tumor interstitial spaces via enhanced permeability and retention (EPR) effect [19].

The morphology of CSH-DTX was investigated using TEM and SEM. The TEM showed a spherical particle with uniform distribution in the copper grid (Figure 2b). The size measured by TEM was smaller than observed via DLS experiment. The discrepancy in size might be attributed to the hydrodynamic state and dried state measurement. The morphology was further confirmed by SEM which showed a smooth regular surface, spherical shaped particles (Figure 2c). The size was consistent with the TEM observation. The drug loading capacity of DTX was observed to be more than 20% with a high entrapment of >95%.

### In vitro drug release

**Figure 3:**
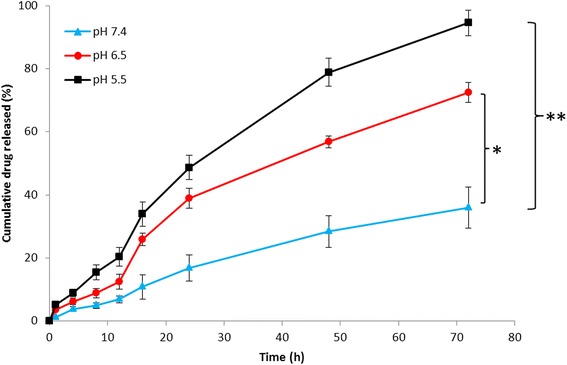
**Release profile of DTX from CSH-DTX micelles incubated at phosphate buffered saline (pH 7.4) and acetate buffered saline (pH 6.8 and 5.0).** The samples were incubated at 37°C in a rotary shaker (100 rpm). The data are presented as mean ± SD (n = 3). *p < 0.05, *p < 0.01 is the statistical difference between drug release at pH 5.0, pH 6.5, and pH 7.4.

The release study was carried out in phosphate buffered saline (PBS, pH 7.4) and acetate buffered saline (ABS, pH 6.8 and pH 5.0). As shown in Figure 3, release rate of DTX from CSH-DTX micelles markedly differed with the change in pH conditions. As expected, accelerated release of DTX was observed at lower pH, while slow release profile was seen at basic pH conditions. At pH 7.4, nearly 30% of drug released while 70% of drug released when the pH of release media was decreased to pH 6.8. Importantly, release rate was further increased when micelles were incubated in pH 5.0 containing media. Nearly 95% of drug released in pH 5.0 at the end of 72 h of study period. In all the pH conditions, although slightly faster release was observed during the initial time points however no burst release pattern was observed. The micelles exhibited a sustained release profile for DTX. It could be expected that at physiological pH conditions, core will be intact and DTX would be blocked in the highly hydrophobic core leading to low release rate. However when the pH decreased, accelerated release was observed due to the protonation of histidine residue. At lower pH, when the histidine was protonated, imbalance of hydrophilic and hydrophobic force destabilizes the micelles structure and the drug diffuses in higher rate [16]. Therefore, CSH micelles could effectively prevent the drug release or drug leakage in physiological (avoids toxicity) conditions while releases rapidly in acidic conditions in response to endosomal and lysosomal pH.

### In vitro cytotoxicity assay

**Figure 4:**
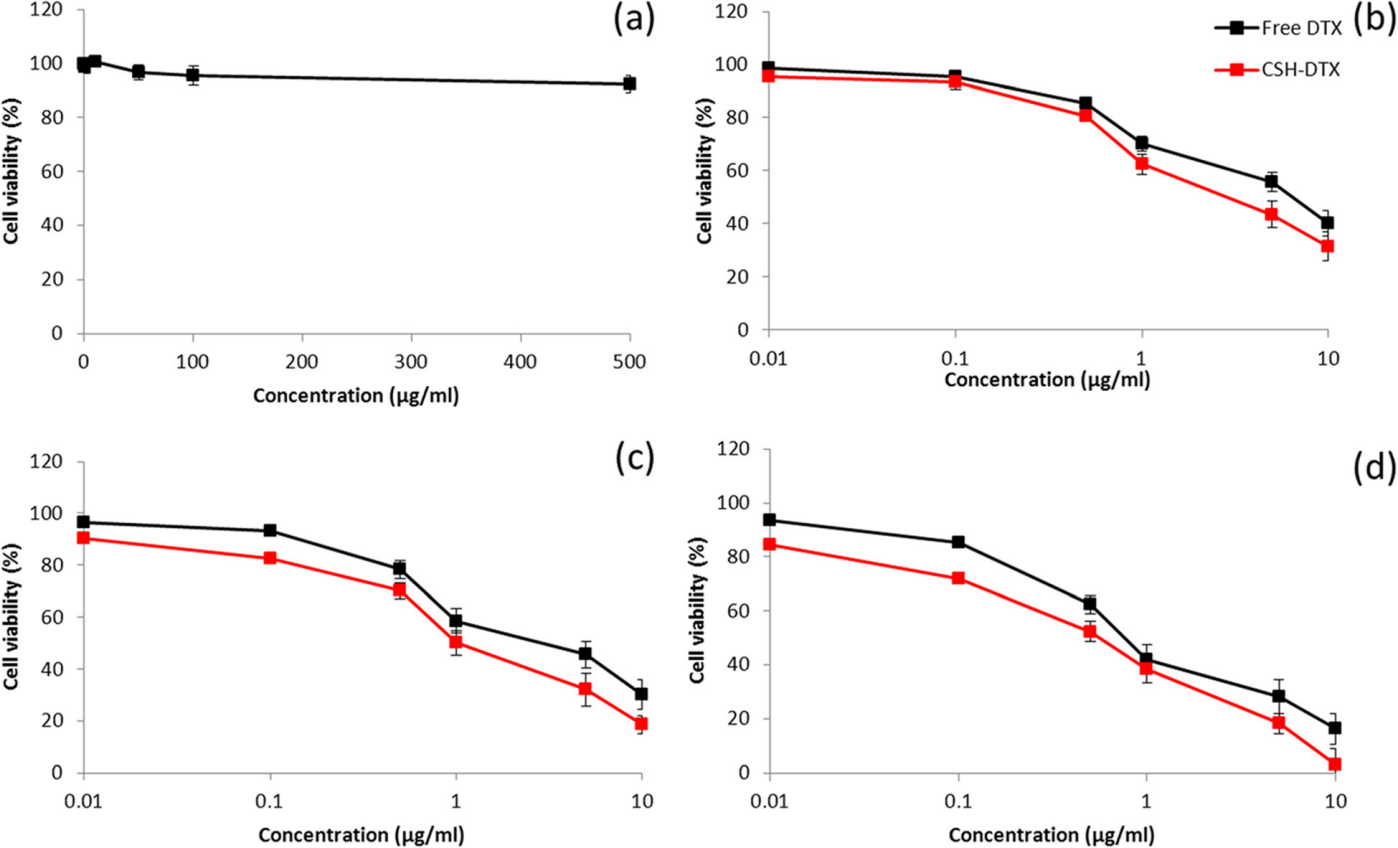
**(a) In vitro cytotoxicity of blank polymeric micelles at various concentrations against QBC939 cells (b-d in vitro cytotoxicity of free DTX and CSH-DTX against QBC939 cells incubated at 24, 48, and 72 h.** The cytotoxicity of formulations was evaluated by MTT assay. The data are presented as mean ± SD (n = 6).

**Figure 5:**
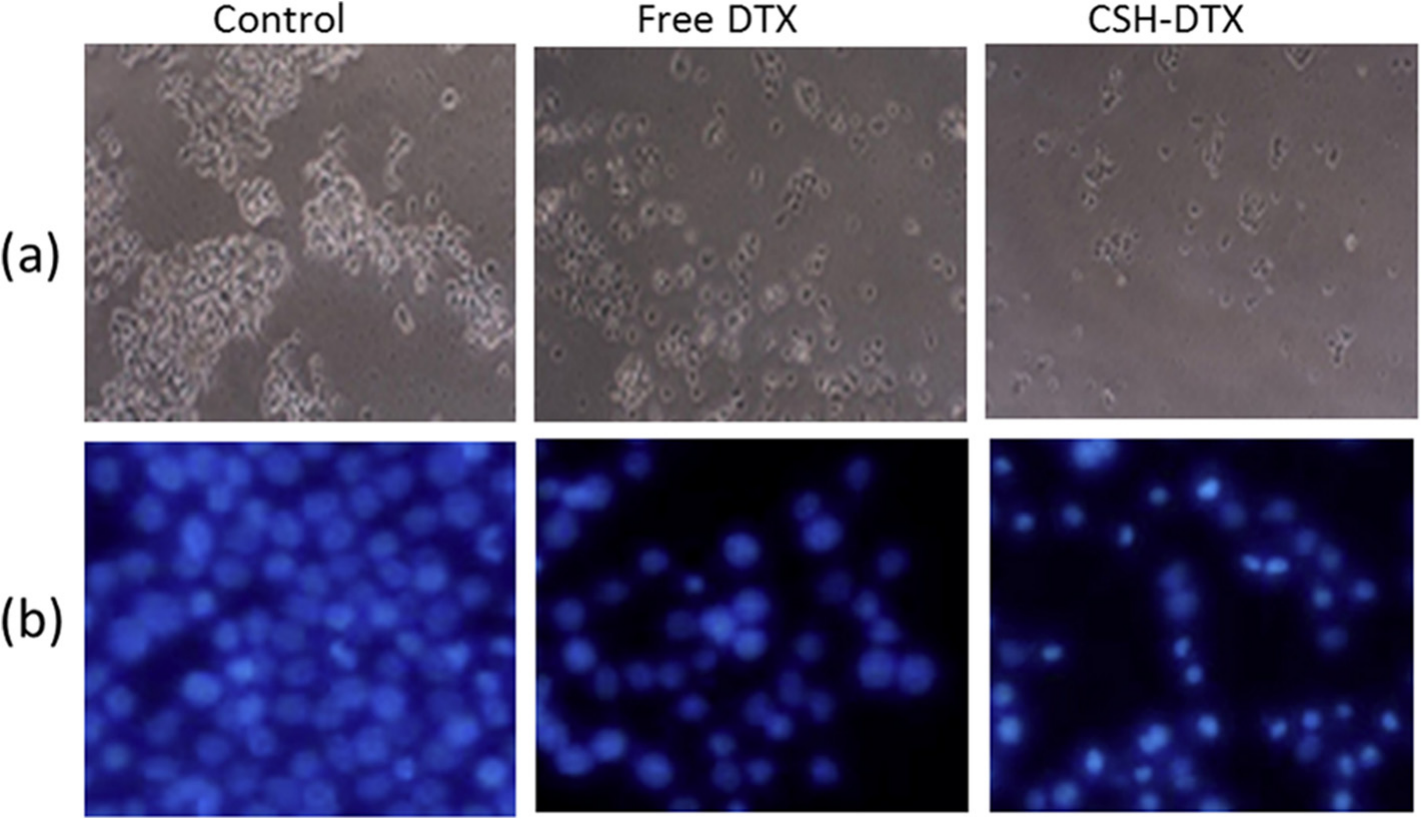
**(a) Cellular morphology of QBC939 cells following incubation with free DTX and CSH-DTX (b) fluorescence microscopy images of the cell apoptosis induced by free DTX and CSH-DTX.** The apoptosis of cells was analysed by Hoechst staining.

The in vitro cytotoxicity of blank copolymer was studied in different concentrations against QBC939 CC cells to evaluate its safety profile. The cells were treated with concentrations between 0.1 μg/ml to 500 μg/ml. As seen (Figure 4a), blank polymeric micelles did not exhibit any significant toxicity in the tested concentration range after 24 h incubation. Especially, cell viabilities remained more than >94% at all the concentrations indicating its excellent safety profile. The least or negligible cytotoxicity of blank polymer makes it ideal for in vivo cancer targeting. Followed by which cytotoxicity of free DTX and CSH-DTX was evaluated in the same cell lines in a concentration and time dependent manner. As shown in Figure 4b-d, both free drug as well as drug loaded micellar formulations exhibited a greater cytotoxicity in a time- and concentration dependent manner. It has to be noted that cytotoxicity of CSH-DTX was more pronounced than that of free drug in all the time points. IC50 value of individual formulation was calculated to quantify the cytotoxic effect. The IC50 value of free DTX remained at 6.45 μg/ml, 2.86 μg/ml, and 0.89 μg/ml after 24, 48, and 72 h incubation, respectively. On the other hand, IC50 value of CSH-DTX stood at 2.58 μg/ml, 0.98 μg/ml, and 0.49 μg/ml for the same time period, respectively. The superior cytotoxicity of CSH-DTX might be attributed to the pH-driven release of active therapeutic molecule in the cell cytoplasm. It could be expected that micelles were internalized into the cells via endocytosis mechanism where in the drug released at acidic compartments and travel to site of action [20]. The cytotoxicity was further confirmed by cellular morphology. As seen Figure 5a, control cells were densely packed on the cover slip and of regular shape, however, DTX treated cells showed signs of apoptosis and cells were round and circular. Importantly, CSH-DTX treated cells were fewer in number (viable cells were decreased) and scattered with a clear sign of membrane blebbing and apoptosis.

### Apoptosis measurements

Changes in cell morphology resulting in the rounding of cells are one of the prominent hallmarks of apoptosis. The apoptosis measurement was carried out by Hoechst 33258 staining. As shown in Figure 5b, untreated cells did not show any changes in morphology and remained same after 24 h. Additionally, cells were densely packed and present large numbers covering the entire cover slip. The free DTX however reduced the number of viable cells and exhibited typical features of apoptosis. Notably, CSH-DTX remarkably induced the apoptosis in cancer cells with typical features of cell death such as chromatic condensation, membrane blebbing and apoptotic bodies were visible. Results indicate that the drug loaded micelles could cause marked condensation and fragmentation of nuclear bodies.

### Apoptosis assay by flow cytometry

**Figure 6:**
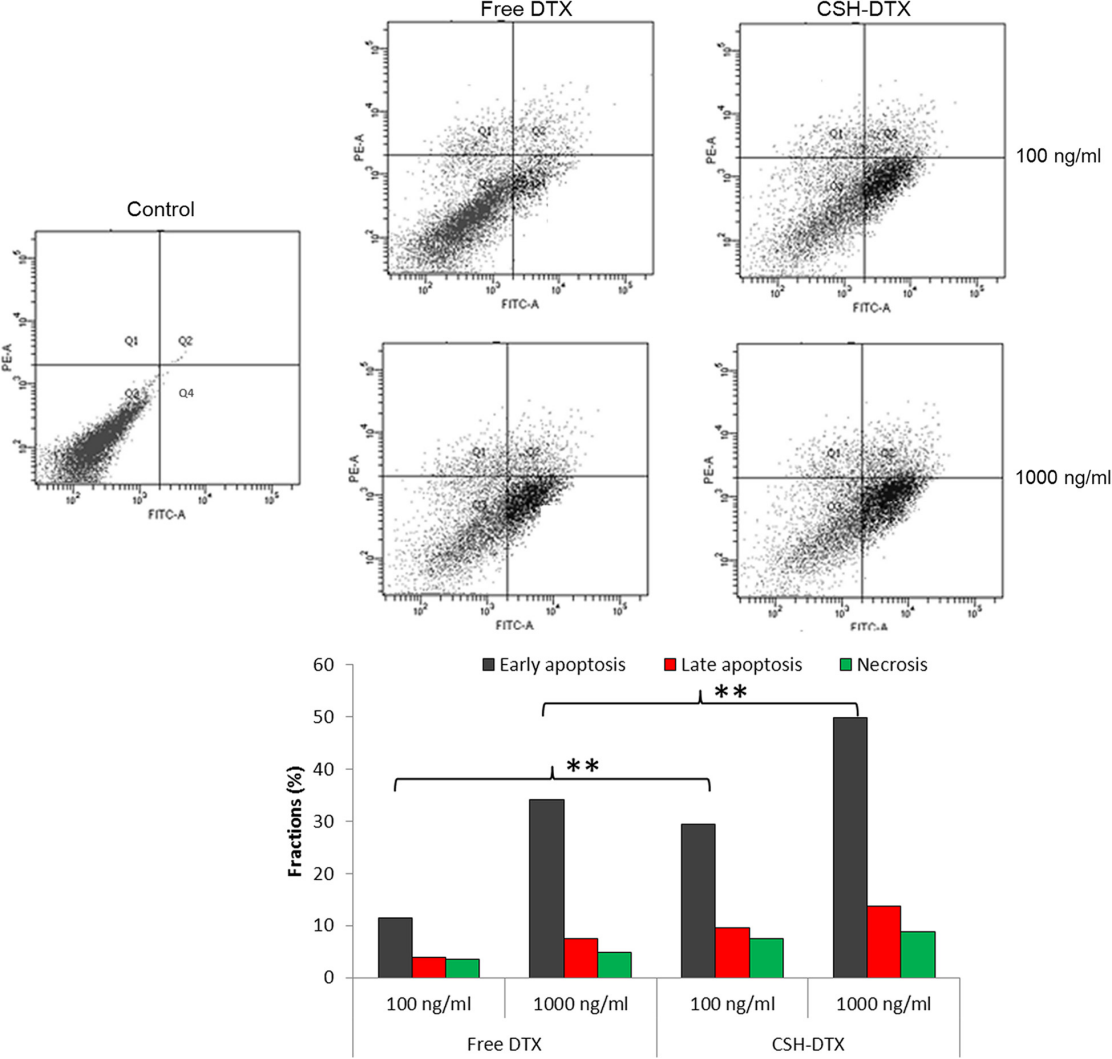
**Flow cytometer analysis of cell apoptosis using annexinV-FITC and PI staining.** The cells were exposed with free DTX and CSH-DTX at a concentration of 100 ng/ml and 1000 ng/ml and incubated for 24 h. **p < 0.01 is the statistical difference CSH-DTX and free DTX.

Figure 6 shows the apoptosis analysis (early and late apoptosis) of QBC939 cells using Annexin V FITC and PI staining by flow cytometer. In the present study, cells were treated with 100 ng/ml and 1000 ng/ml of free DTX and equivalent CSH-DTX formulations and incubated for 24 h. Results indicate that the proportion of early and late apoptosis cells markedly increased with the increase in the concentration of chemotherapeutic drugs. For example, ~10% of cells were in early apoptosis quadrant when exposed with 100 ng/ml of free DTX, while it increased to ~32% for exposure to 1000 ng/ml of drug. As expected, CSH-DTX resulted in higher apoptosis of cancer cells with ~30% and ~50 of cells in early apoptosis quadrant for the same concentrations, respectively. Similarly, late apoptosis cells increased as well in a concentration-dependent manner. The result was consistent with the cytotoxicity that micellar formulation could remarkably induce the cell apoptosis.

### In vivo antitumor efficacy

**Figure 7:**
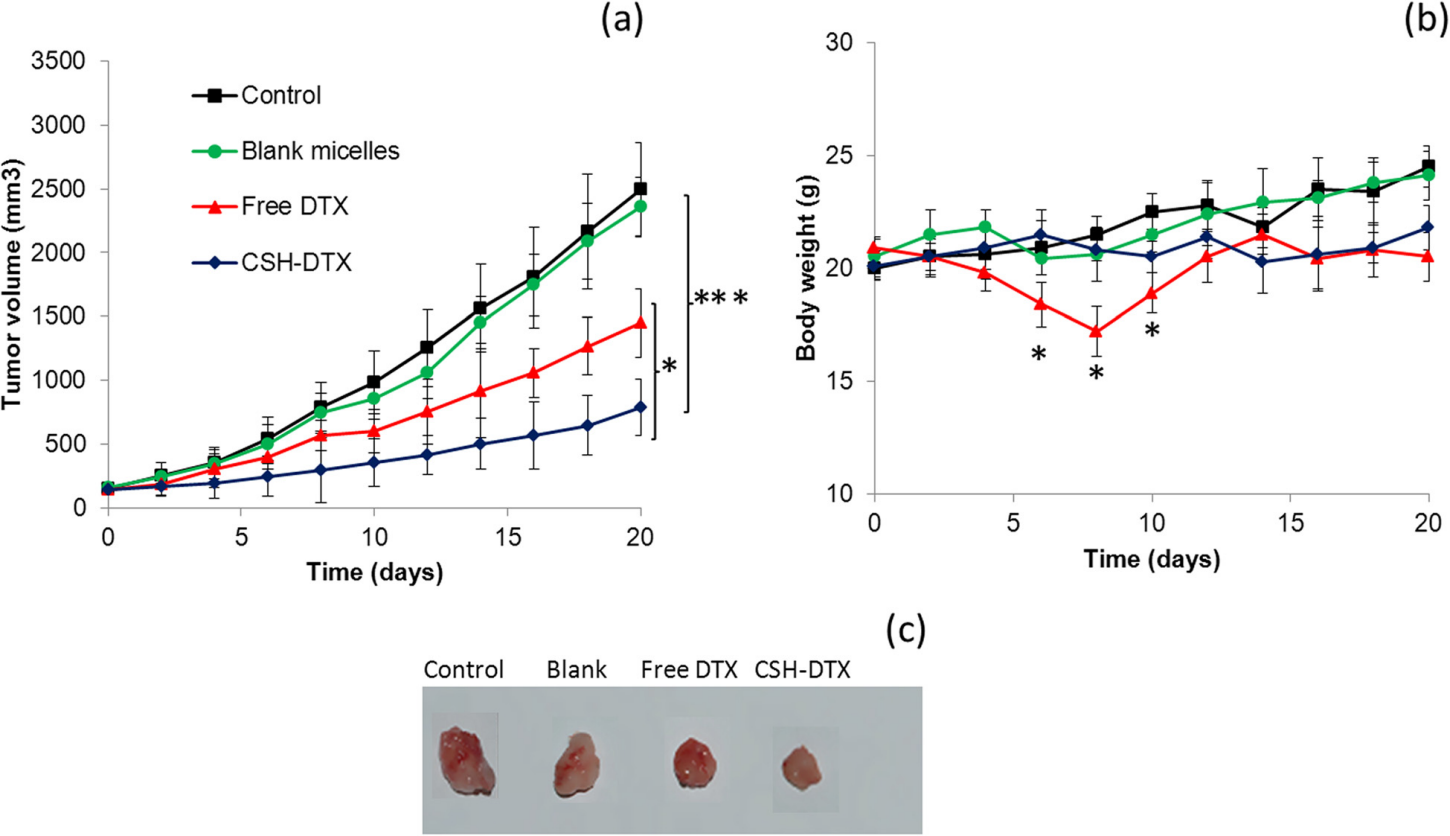
**In vivo antitumor efficacy study (a) changes in tumor volume (b) changes in mice body weight (c) images of tumor sections.** The antitumor study was carried out in QBC939 cells -bearing xenograft model and administered thrice at a fixed dose of 5 mg/kg. *p < 0.05, ***p < 0.001 is the statistical difference in the tumor volume between CSH-DTX and free DTX or CSH-DTX and control group.

The antitumor efficacy of free DTX and CSH-DTX was investigated in QBC939 cells bearing xenograft tumor model. The mice were intravenously injected with respective formulations every 3^rd^ day for three times. The tumor volume and body weight was noted every alternative day up to day 20. As shown in Figure 7a, CSH-DTX significantly slowed down the growth of tumor in mice models comparing to that of free DTX and saline treated mice groups. As expected, blank micelles did not have any effect on the tumor volume of mice and grew along with control group. Administration of free DTX although showed some therapeutic effect however could not inhibit it’s growth completely. The micellar formulations remarkably suppressed the tumor proliferation by comparison to that of control and free drug treated animal group. The final tumor volume of control, blank micelles, free DTX and CSH-DTX treated groups were ~2500, ~2500, ~1300, and ~600 mm^3^, respectively. The main reason behind the superior antitumor efficacy of CSH-DTX was attributed to increased accumulation of micelles in tumor regions due to EPR effect and enhanced sensitization of MDR cancer to DTX. The other reasons might be due to the sustained release of drug and prolonged blood circulation [21].

Along with therapeutic efficacy of anticancer drug loaded delivery system, minimization of side effects remains a big challenge for the successful cancer chemotherapy. The change in body weight has been considered to be an index to evaluate the systemic toxic effects. As shown in Figure 7b, mice group treated with free DTX significantly reduced its body weight. Approximately, 20% of body weight was reduced in this group indicating its systemic toxicity. On the other hand, when the same dose of drug was loaded in polymeric micelles, no body weight-loss was observed and throughout the study period the body weight was stable. It should be noted that the body weight of DTX treated group started recovering approximately after 8 days (after final injections). The body weight recovery might be due to the slow removal of free drug from the vital organs and clearance from the systemic circulation. The result therefore indicates that CS-HS based micelles effectively reduced the drug related side effects while at the same time improved its therapeutic efficacy as shown by reduced tumor volume [22].

### Histopathological and immunohistochemical analysis

**Figure 8:**
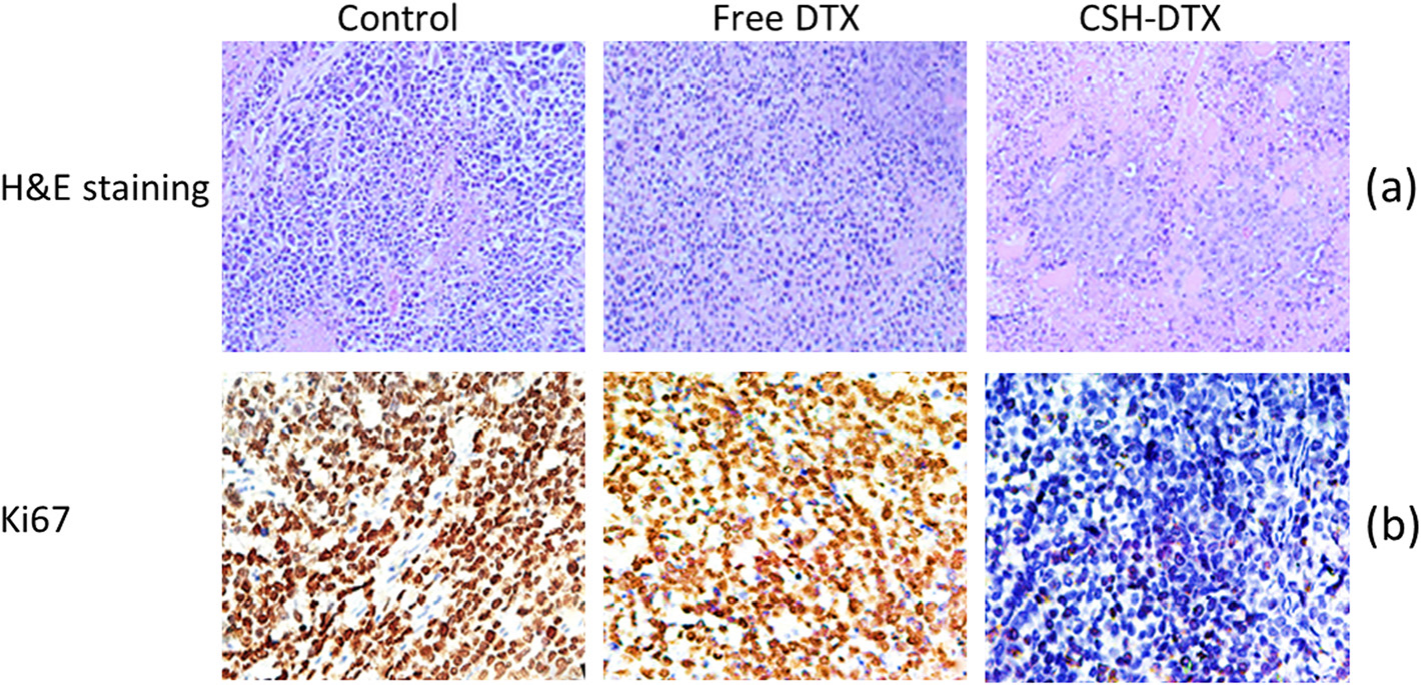
**(a) histopathology of tumor sections (b) immunohistochemical analysis of tumor cell proliferation (Ki-67) (c) immunohistochemical analysis of cleaved PARP (apoptosis marker).**

H & E staining was performed to stain the tumor sections wherein nucleus was stained with hematoxylin (blue) and extracellular matrix was stained with eosin (pink). As shown in Figure 8a, control group exhibited clear cell morphology with excess chromatin and binucleolates. Whereas, free DTX treated group showed range of necrosis with irregular cellular morphology. The tissue necrosis further increased for CSH-DTX treated group with distinct damage to cancer cells. Lack of nuclei and lack of boundary regions were observed in this group.

Immunohistochemical staining of Ki-67 was performed to evaluate the tumor proliferation ability of individual formulations. As seen (Figure 8b), fewer number of Ki-67 cells were present in CSH-DTX treated cells comparing to that of free DTX treated group. This further confirms the enhanced accumulation of drug in the tumor tissues from micellar formulations and enhanced sensitization of MDR cancer to DTX.

PARP, a DNA binding enzyme is cleaved by caspase-3 and caspase-7. PARP is an important indicator of apoptosis in cancer cells. In this study, level of PARP was considered as a marker for level of cell apoptosis. As shown in Figure 8c, cleaved PARP was detected in DTX treated group, while it was more significant in CSH-DTX treated group. The enhanced apoptosis in CSH-DTX treated group was consistent with its excellent antitumor efficacy.

## Conclusions

An amphiphilic block copolymer CS-HS-based polymeric micelles was prepared and loaded with DTX to target cholangiocarcinoma. The pH-sensitive behaviour of histamine in the block copolymer will accelerate the release of DTX in the tumor region while protects the therapeutic load in the physiological conditions. In the present study, CSH-DTX controlled the release of drug in the basic pH while rapidly released its cargo in the tumor pH (pH 5 and 6.8) possibly due to the breakdown of polymeric micelles. A nanosize of <150 nm will allow its accumulation in the tumor interstitial spaces via EPR effect. CSH-DTX effectively killed the cancer kills in a time- and concentration-dependent manner and showed pronounced therapeutic action than that of free drug at all-time points. The superior cytotoxicity of CSH-DTX might be attributed to the pH-driven release of active therapeutic molecule in the cell cytoplasm. CSH-DTX resulted in higher apoptosis of cancer cells with ~30% and ~50 of cells in early apoptosis quadrant when treated with 100 and 1000 ng/ml of equivalent drug. The micellar formulations showed remarkable effect in controlling the tumor growth and reduced the overall tumor volume to 1/5^th^ to that of control and half to that of free drug treated group with no sign of drug-related adverse effects. Immunohistochemical analysis of tumor sections showed that fewer number of Ki-67 cells were present in CSH-DTX treated cells comparing to that of free DTX treated group. Our data suggests that nanoformulation of DTX could potentially improve the chemotherapy treatment in cholangiocarcinoma as well as in other malignancies.

## Materials and methods

### Materials

Docetaxel was procured from Sigma-Aldrich (China). Chondroitin sulphate (CC) was procured from Shanghai Sangon Biological Engineering Technology & Services Co. Ltd. (Shanghai, China). Histamine dihydrochloride (HS) was purchased LSB Biotechnology Inc. (Xi’an, China). 1-(3-(Dimethylamino)propyl)-3-ethylcarbodiimide hydrochloride (EDC), N hydroxysuccinimide (NHS) was obtained from Sigma-Aldrich (China). All other chemicals were of reagent grade and used without further purification.

### Synthesis of chondroitin sulphate (CS)-histamine (HS) conjugate

Chondroitin sulphate was conjugated with histamine as reported previously [23]. Briefly, CS was dissolved in 120 ml of phosphate buffered saline (PBS, pH 6.0) maintained in a magnetic stirrer for 5 h. Carboxyl group of CS was activated by the addition of EDC and NHS in specific quantity one by one. After 30 min, HS was added and allowed the reaction mixture to proceed for 24 h. The resultant reaction mixture was dialyzed against phosphate buffer and then the process was repeated with distilled water. Finally, the product was lyophilized and stored in dark place.

### Preparation of docetaxel-loaded polymeric micelles

DTX-loaded CS-*b*-HS micelles (CSH-DTX) were prepared by a solvent extraction and evaporation method. Briefly, specific quantity of DTX and CS was dissolved in 10 ml of dichloromethane. This organic solution was poured in distilled water and immediately sonicated for 5 min to form an O/W emulsion system. The reaction was allowed to proceed for 24 h in the dark conditions.

### Particle size and zeta potential analysis

The mean diameter and surface charge was analyzed using dynamic light scattering technique by Zetasizer (Nano-ZS 90, Malvern, Worcestershire, UK). The samples were measured at 25°C at a fixed angle of 90°C. Each sample was measure in triplicate.

### Morphology analysis

The morphological examination of nanoparticles was carried out using transmission electron microscope (TEM) (JEM-2010; JEOL, Japan). Nanoparticle dispersion was placed on the carbon-coated copper grid and negatively stained with 2% (w/v) phosphotungstic acids and air dried. The morphology and surface texture was further confirmed by scanning electron microscopy (SEM; FEI Nova NanoSEM 230). The samples were freeze dried and coated with platinum before the SEM analysis.

### Drug-loading and encapsulation efficiency

UV–vis Spectrophotometer was used to calculate loading capacity and entrapment efficiency of DOX in CSH micelles was estimated by HPLC technique (LC 1200; Agilent Technologies, Santa Clara, CA, USA). The mobile phase consists of acetonitrile and 0.2% trimethylamine (pH adjusted to 6.4 with phosphoric acid) (48:52, v/v) at flow rate of 1 mL/min.

The dried solid samples were dissolved in 1 ml of dichloromethane and sonicated vigorously for 10 min. This solution was centrifuged (20000 rpm) and supernatant was collected and injected into HPLC column. A reverse-phase C18 column (250 mm × 4.6 mm; GL Science, Tokyo, Japan) was used. The mobile phase was run at 1 ml/min and detected at 254 nm.

$$ \mathrm{D}\mathrm{L}\%=\frac{\mathrm{Total}\;\mathrm{Drug}\kern0.24em \mathrm{added}}{\mathrm{Wt}.\kern0.5em \mathrm{of}\;\mathrm{P}\mathrm{olymer}\kern0.5em +\kern0.5em \mathrm{W}\mathrm{t}.\kern0.5em \mathrm{of}\;\mathrm{drug}\;\mathrm{in}\;\mathrm{N}\mathrm{P}}\kern0.5em \times \kern0.5em 100\% $$

$$ \mathrm{E}\mathrm{E}\%=\frac{\mathrm{Actual}\;\mathrm{drug}\;\mathrm{loading}}{\mathrm{Theoretical}\;\mathrm{drug}\;\mathrm{loading}}\kern0.5em \times \kern0.5em 100\% $$

### Drug release study

The drug release study was carried out in various pH medium. For the release study, freeze dried micelles were reconstituted in distilled water and 1 ml of it was placed in dialysis tube and clipped at both the end. The dialysis tube was placed in a falcon tube with 30 ml of release media (different pH level). The whole assembly was placed in a shaking bath at 37°C. At predetermined time intervals, 1 ml of release sample was collected and replaced with equal amount of fresh release media. The amount of drug released in the release media was calculated from the HPLC technique.

### Cell culture

QBC939 cholangiocarcinoma cells were cultured in RPMI 1640 medium supplemented with 10% fetal bovine serum (FBS) and 1% penicillin streptomycin mixture. The cells were maintained in ambient conditions of 37°C and 5% CO_2_.

### Cytotoxicity assay

The cytotoxicity assay was measured by 3-(4,5-dimethythiazol-2-yl)-2,5-diphenyl tetrazolium bromide (MTT) assay. It is based on the reduction of yellow MTT by mitochondrial succinate dehydrogenase. MTT enters the live cells and reduced into insoluble formazan complex. For this, QBC939 cells were seeded at a density of 1 × 10^4^ in a 96-well plate. After 24 h, cells were exposed to blank polymer, free DTX and CSH-DTX at different dosing level. The cells were incubated for 24, 48 and 72 h accordingly. At each time point, plate was removed and treated with 100 μl of MTT solution (5 mg/ml) to each 96-well plate and incubated for 4 h. The formed formazan crystals were extracted by adding DMSO and incubated for additional 30 min. The absorbance of each plate was read at 570 nm using a microplate reader (Thermo-Fisher, USA). All experiments were repeated 6 times.

The morphology of cells was observed using a fluorescent microscope (Leica DM IRBE microscope) and representative images were selected.

### Apoptosis measurement

Hoechst 33258 was used to observe the cell apoptosis. During cell apoptosis, condensation of chromatin takes place and DNA gets cleaved into small fragments. Generally, it enters the live cells and binds with adenosine-thymidine (AT) part of DNA while in apoptotic cells, it binds to condensed chromosome. Normal cells and apoptotic cells were different in their size and distinct morphology. The drug treated cells were washed with PBS and stained with Hoechst 33258 for 10 min. The cells were washed and fixed with 4% paraformaldehyde and observed under fluorescence microscope.

### Apoptosis analysis by flow cytometry

Apoptosis assay was carried out by flow cytometer. For this, cells were seeded, incubated for 24 h and treated with respective formulations (Free DTX and CSH-DTX). The treated cells were further incubated for 24 h. The cells were harvested and washed with PBS. The pellets were resuspended with 100 μl of binding buffer (10 mM HEPES pH 7.4, 150 mM NaCl, 5 mM KCl, 1 mM MgCl_2_, and 1.8 mM CaCl_2_). The cells were then treated with FITC-Annexin V and incubated for 20 min and then PI was added and incubated for additional 10 min. The cells were analysed for apoptotic cells using FACS (Becton Dickenson Biosciences, San Jose, CA, USA).

### In vivo antitumor efficacy study

In vivo antitumor efficacy study was performed in 7-week old xenograft nude mice. Briefly, 1 × 10^6^ QBC939 cells (100 μl PBS) were subcutaneously injected into the right flank of nude mice to establish cholangiocarcinoma tumor models. The tumours were allowed to grow for two weeks until it reaches ~150 mm^3^ size. The mice were equally divided into 4 groups with 8 mice in each group; untreated controls, blank micelles, free DTX, and CSH-DTX at a fixed dose of 5 mg/kg. The samples were injected thrice via tail vein injection during first two weeks. The tumor size was measured using Vernier calliper for every other alternative day. Tumor volume was calculated using the formula: volume = 1/2 × D_max_ × (D_min_)^2^. The body weight was measured simultaneously as an indicator of the systemic toxicity. At the end of the study period, tumors were surgically removed and fixed in 10% neutral formalin and embedded in paraffin.

### Histopathological and immunohistochemical evaluations

The histopathology of tumor sections was evaluated by hematoxylin and eosin (H & E) method. The embedded paraffin tumor sections were cut into 5 μm slices and stained with H & E staining agent and viewed by microscope (Nikon TE2000U). For immunohistochemical analysis, rabbit monoclonal primary antibody for cleaved poly-ADP-ribose polymerase (PARP) (Abcam, Cambridge, MA, USA) and rat anti-mouse Ki-67 monoclonal antibody (Maixin Biotechnology Co., Ltd) to quantify Ki-67 expression was used in the study.

### Statistical analysis

The experimental data are presented as the mean (standard deviation (SD). All statistical analyses were performed using ANOVA or a two-tailed Student’s t-test (GraphPad Prism 5).

## Abbreviations

HS: Histidine
CS: Chondroitin sulphate
NP: Nanoparticles
DTX: Docetaxel
